# Proteomic Characterization of the Venom of Five *Bombus* (*Thoracobombus*) Species

**DOI:** 10.3390/toxins9110362

**Published:** 2017-11-11

**Authors:** Nezahat Pınar Barkan, Mustafa Bilal Bayazit, Duygu Ozel Demiralp

**Affiliations:** 1 Department of Biology, Faculty of Science, Hacettepe University, 06800 Ankara, Turkey; pinar.barkan@gu.se; 2 Department of Fundamental Neurosciences, University of Lausanne, 1005 Lausanne, Switzerland; mbayazit@unil.ch; 3 Faculty of Engineering, Biomedical Engineering, Ankara University, 06830 Ankara, Turkey

**Keywords:** bumble bees, toxins, MALDI-TOF MS, proteomics, 2D-PAGE, venom

## Abstract

Venomous animals use venom, a complex biofluid composed of unique mixtures of proteins and peptides, to act on vital systems of the prey or predator. In bees, venom is solely used for defense against predators. However, the venom composition of bumble bees (*Bombus* sp.) is largely unknown. The *Thoracobombus* subgenus of *Bombus* sp. is a diverse subgenus represented by 14 members across Turkey. In this study, we sought out to proteomically characterize the venom of five *Thoracobombus* species by using bottom-up proteomic techniques. We have obtained two-dimensional polyacrylamide gel (2D-PAGE) images of each species’ venom sample. We have subsequently identified the protein spots by using matrix assisted laser desorption ionization/time-of-flight mass spectrometry (MALDI-TOF MS). We have identified 47 proteins for *Bombus humilis*, 32 for *B. pascuorum*, 60 for *B. ruderarius*, 39 for *B. sylvarum*, and 35 for *B. zonatus*. Moreover, we illustrated that intensities of 2DE protein spots corresponding to putative venom toxins vary in a species-specific manner. Our analyses provide the primary proteomic characterization of five bumble bee species’ venom composition.

## 1. Introduction

In venomous animals, the use of venom or poison is one of the most essential mechanisms for either capturing prey or defending oneself against predators. In competing for resources, venom is an adaptive trait and an example of convergent evolution. It is a complex biofluid secreted by the venom gland and is composed of a unique mixture of proteins, enzymes, peptides, and biogenic amines that act on vital systems of the recipient [1]. In real bees (Apoidea), the largest family of bees with over 20,000 members [2], venom is solely used for defense against predators [3].

Among Apoidea, the majority of venom studies are focused on the honey bee *Apis mellifera* (*A. mellifera*). *Bombus* sp., which also belongs in the same family as *A. mellifera*, are pollinators for various native and cultivated plants [4]. The venom composition of *Bombus* sp. is poorly understood.

Previous studies on *Bombus* sp. venom have mainly focused on characterization and isolation of highly abundant single molecules using bio-assays and chemical sequencing via Edman degradation [5,6,7,8,9,10,11,12,13,14,15,16]. Among the described peptides, bombolitins are unique to *Bombus* sp. and enhance phospholipase A_2_ in liposome hydrolysis, in a similar fashion to melittin found in *A. mellifera* venom and crabrolin and mastoparan found in vespid *Vespa crabro* venom [17]. Phospholipase A_2_ [18,19,20], serine proteases [6,8,10,11,13,20], serine protease inhibitors [14,15], acid phosphatases [18,19,20], arginine kinase [21], hyaluronidase [19], putrescine [22], citrate [23], defensin [24], and acethylcoline [25] have also been isolated from *Bombus sp*. venom. 

Recently, the whole genome sequencing of European large earth bumblebees *B.* (*Bombus*) *terrestris* and *B. impatiens* have become publicly accessible [26]. The accessibility to the genome sequence, combined with the use of mass spectrometry, paved the way for deeply analyzing the venom proteome composition of *B. terrestris* [20]. This study has identified 55 new molecules in the *B. terrestris* venom. Moreover, 72% of identified molecules had homologs in *A. mellifera* venom, indicating that while there are some species-specific differences, these two bee species share major defense mechanisms. *B. terrestris* is the only member of the *Bombus* sp. whose venom proteome composition has been characterized. 

In bumble bees, color patterns show similarity among species or are highly variable within species. Therefore, classification based on coat color can be unreliable [27]. This is especially true for the *Thoracobombus* subgenus, which consists of 14 species in Turkey that are morphologically difficult to separate. To overcome this, we have previously used landmark-based geometric morphometrics on their wing structure [28]. Other approaches, such as the use of pheromone analysis [29,30,31,32] and DNA-based molecular techniques [33,34,35,36,37,38,39] have also been used in differentiating bumble bee species. 

Turkey is one of the countries with the highest number and diversity of bumble bee species in the in the West-Palearctic region [28]. The main aim of this study is to determine and compare the venom protein profiles of different *Thoracobombus* species in Turkey using bottom-up proteomic strategies. With the adoption of these approaches, not only will we characterize the venom proteome of the *Thoracobombus* species for the first time, but we will also highlight the proteomic differences within the subgenus that may help in differentiating between the species.

## 2. Results

### 2.1. 2D-PAGE Analysis of Thoracobombus Venom

The 2DE gel images were obtained from each venom sample (Figure S1). 

In the *Thoracobombus* match set, 87 protein spots were manually matched in 2DE images of *B. humilis*, *B. pascuorum*, *B. sylvarum*, *B. ruderarius*, and *B. zonatus* venom. Spot quantities were calculated and significant spots were detected statistically. Among these protein spots, 21 spots were present in all species. Common and differentially expressed protein spots are summarized (Table S2). 

### 2.2. Mass-Spectrometry of Thoracobombus Venom

**Figure 1:**
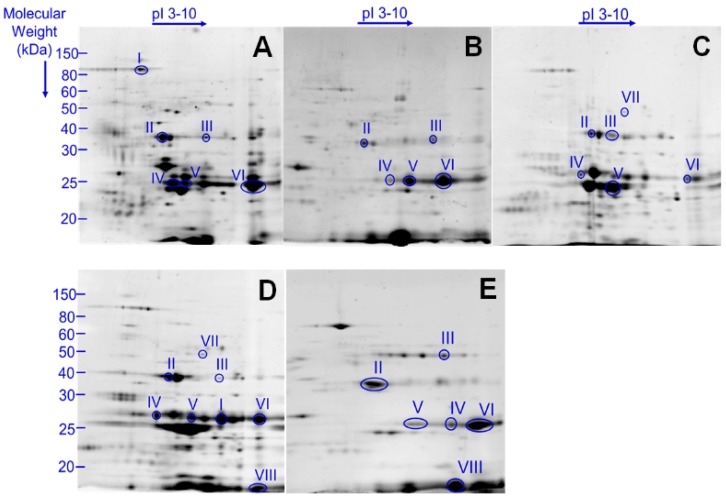
2DE gel images of *Thoracobombus* venom. Representative images of venom profile of (**A**) *B. humilis*; (**B**) *B. pascuorum*; (**C**) *B. ruderarius*; (**D**) *B. sylvarum*; (**E**) *B. zonatus*. Gel analyses were conducted in triplicates. Putative venom toxins marked with roman numerals: I: Peptidase I *, Aminopeptidase N **; II: Venom acid phosphatase Acph1-like; III: Hyaluronidase 1; IV: Venom protease; V: Arginine Kinase; VI: Phospholipase A_2_; VII: Serine protease snake; VIII: Kunitz-type serine protease inhibitor. (See Table 1).

Using 2DE gel images, we have spectrometrically characterized 47 proteins for *B. humilis*, 32 for *B. pascuorum*, 60 for *B. ruderarius*, 39 for *B. sylvarum*, and 35 for *B. zonatus* (Figure S1 and Tables S3–S7). Putative toxins are categorized according to their function and subcellular location [20] (Figure 1). Toxins previously identified in *B. terrestris* and *A. mellifera* venoms are used as references (Table 1) [20,40]. Major putative toxins phospholipase A_2_, venom protease, venom acid phosphatase, and hyaluronidase were detected in venom of all five analyzed *Thoracobombus* species. Arginine kinase, a known component of spider and parasitoid wasp venom, was also identified in all studied species. In contrast, serine protease snake (*B. ruderarius* and *B. sylvarum*), peptidases (*B. humilis* and *B. sylvarum*), and kunitz-type serine protease inhibitor (*B. humilis* and *B. sylvarum*) were only detected in certain species.

### 2.3. Species Specific Expression of Putative Venom Toxins

**Figure 2:**
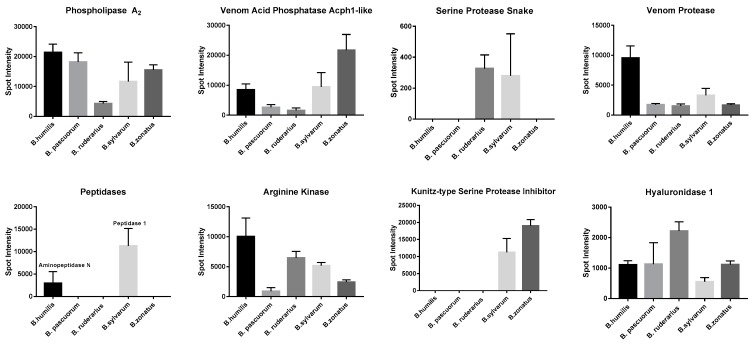
Relative 2DE gel spot intensities of putative toxins in *Thoracobombus* venom.

Next, we compared the intensities of 2DE gel spots corresponding to putative toxins in each sample. Several toxins displayed species-specific expression patterns (Figure 2). The expression of phospholipase A_2_, was the lowest in *B. ruderarius* venom among the studied species. *B. zonatus* venom displayed the highest venom acid phosphatase expression. *B. pascuorum* and *B. ruderarius* were characterized by a relatively low venom acid phosphatase expression. *B. humilis* and *B. pascuorum* displayed the most and the least abundant arginine kinase profile, respectively. *B. humilis* displayed the highest venom protease expression. Hyaluronidase 1 expression was more than two folds higher in *B. ruderarius* venom, compared to the other species.

Intensities, as calculated by PDQuest Software, are presented as mean ± standard deviation (3 replicates).

## 3. Discussion

Identification of the *Thoracobombus* subgenus represents a challenge as its members are morphologically similar. In our previous study, we had applied landmark-based geometric morphometrics to distinguish its members from each other more accurately. In the current study, we provide the primary proteomic characterization of the venom profile of five bumble bee species belonging to this subgenus. Several putative venom toxins are detected in *Thoracobombus* which are in accordance with *B. terrestris* and *A. mellifera* venom. Furthermore, we outline the species-specific expression patterns of putative toxins. 

The defensive behavior of social groups of Hymenoptera is similar to each other. Venom is used for protecting the colonies rather than for capturing prey. Therefore, the venom compositions of these groups are expected to have common overall pattern. It is illustrated that the venom profile of *B. terrestris* and *A. mellifera* largely overlap, while also retaining certain species-specific differences. 

Accordingly, in *Thoracobombus* venom, we have identified putative venom toxins collectively used by several Hymenoptera species to debilitate predators such as phospholipase A_2_, arginine kinase, acid phosphatase (venom acid phosphatase Acph-1), serine protease (serine protease snake), peptidases (peptidase 1 and aminopeptidase N), hyaluronidase (hyaluronidase 1), and serine protease inhibitors (kunitz-type serine protease inhibitor). 

While the *Thoracobombus* venom composition may be similar to other Hymenoptera species in terms of major toxins identified, it is important to note that the venom may be tailored for the individual species’ needs with regards to abundancies of protein families present. Accordingly, a change in the protein family profile of snake venom has been described upon nutritional shift [41]. 

Here, we have compared the 2DE gel images obtained from different *Thoracobombus* species and highlighted the species-specific expression differences of venom proteins. In particular, our results indicate that expression levels of putative toxins may be used in differentiating between species. Within the *Thoracobombus* subgenus, *B. humilis* is morphologically very similar to *B. pascuorum*, while *B. ruderarius* and *B. sylvarum* resemble each other in terms of morphology and coat color. On the other hand, *B. zonatus* with its characteristic yellow coat color is easily distinguished from others. We have collected *B. ruderarius* and *B. sylvarum* at the highest altitudes (>2000 m) and *B. zonatus* at the lowest (~1000 m). Interestingly, we have found that morphologically similar species differentiate in their expression of putative venom toxins, even in a similar habitat. 

In future studies, different populations of same species can be used to assess the direct effect of altitude, vegetation, and prey population on the venom composition of bees. One of the main limitations of this study is the lack of genome sequences of the studied species, which hinders the database matching for analyzed samples. In this study we utilized 2DE gel images and MALDI-TOF mass spectrometry for assessing the expression levels of putative toxins, which may have its disadvantages. For example, we could not detect bombolitin, unique bumble bee peptides, in our samples. Bombolitins are very small peptides (10 kilo Daltons), and we may have failed to detect bombolitins and other very small peptides with our equipment. More sensitive quantitative mass-spectrometry approaches, such as liquid chromatography (LC-MS/MS), fragmentation- and label-free quantitation of the corresponding protein may be necessary to increase the sensitivity of the study and confirm the correlation between the microenvironment and the venom profile of bumble bees. 

Profiling bumble bee venom can have pharmaceutical benefits. Venom peptides can be orally active and transported through the blood–brain barrier and through mammalian cell membranes. Commercial venom-derived drugs are available for the treatment of major diseases such as diabetes, stroke, and hypertension [42]. Bumble bee venom may have the potential to serve as an alternative source of these drugs, which are mostly snake venom-derived. Understanding the species-specific expression patterns of toxins can also benefit the specific-compound-oriented pharmacological analyses. 

In conclusion, we provide the primary proteomic characterization of a diverse bumble bee subgenus and illustrate species-specific differences in toxin expression.

## 4. Methods

### 4.1. Venom Extraction

*B. humilis* (Illiger), *B. pascuorum* (Scopoli), *B. ruderarius* (Müller), *B. sylvarum* (Linnaeus), and *B. zonatus* (Smith) were collected from the Ankara, Bolu, Çankırı, and Kayseri provinces from the Middle Anatolian Region during June, July, August, and September in 2013 and 2014 (Table S1), with permission from the Republic of Turkey Ministry of Forestry and Water Affairs Directorate of Nature Conservation & National Parks and the Republic of Turkey Ministry of Food, Agriculture & Livestock. Live specimens were transported to the Hacettepe University Department of Biology for venom extraction. For venom extraction, the venom reservoir was removed from the stinger in a sterile environment with forceps and the membrane was disrupted afterwards. From each individual, this method yielded 2–5 µL of venom, which was collected in a cryotube and transported in liquid nitrogen. Venom samples belonging to the same species were pooled and stored at −80 °C until use. 

### 4.2. Proteomic Analysis

#### 4.2.1. Protein Quantification and Two-Dimensional Gel Electrophoresis

Venom of the aforementioned bumble bee species was collected from 20 individuals per species. Protein concentrations of the venom samples were obtained by using the Bradford assay [43]. Venom was dissolved in 20 µL rehydration buffer [7 M urea, 2 M thiourea, 4% CHAPS (*w*/*v*), 1% ampholytes (pH 3–10), 10 mM DTT, and trace amount of bromophenol blue]. Two-dimensional polyacrylamide gel (2D-PAGE) was performed in three technical replicates as previously described [44] and a total of 50 µg of protein was loaded per gel. For minimizing the –SH oxidation of proteins responsible for the vertical and horizontal tailing in 2D gels, alkylation and reduction were performed by equilibrating IPG strips in equilibration buffer I (6 M urea, 0.375 M Tris-HCl, 2% SDS, 20% glycerol, 2% DTT) and equilibration buffer II (6 M urea, 0.375 M Tris-HCl, 2% SDS, 20% glycerol, 2.5% Iodoacetamide, bromophenol blue).

#### 4.2.2. 2D-PAGE Analysis

The 2D gel electrophoresis (2DE) gels from venom samples of 5 different *Thoracobombus* species were stained with OrioleTM Fluoroscent Gel Stain (Bio-Rad, Munich, Germany). The gels were analyzed using PDQuest Software Advanced 8.0.1 (Bio-Rad, San Diego, CA, USA). The PDQuest software examines each spot on every gel image and picks a master gel containing most spots. All spots in each gel image are then matched onto the master gel. Each post is annotated with an identification number (SSP). For quantifying spots, four different normalization options are performed, including total density in gel images, local regression, total quantity in valid spot, and mean of log ratios. In the present study, 2DE image analysis is performed to both detect common and/or unique spots between samples and quantify spot intensity, which accounts for the amount of protein in that spot. All spots were matched manually and ‘total density in gel images’ normalization was performed. If the same putative toxin was identified in different spots on the gel, then the spot with the highest score and proximity to theoretical pI/mW was chosen for analyses. Two-way ANOVA was performed to detect significant differences between spot intensities (IBM Corp. Released 2010. IBM SPSS Statistics for Windows, Version 19.0, Armonk, NY, USA).

#### 4.2.3. In-Gel Enzymatic Digestion, Matrix-Assisted Laser Desorption Ionization/Time of Flight (MALDI-TOF) Mass Spectrometry and Peptide Mass Fingerprinting (PMF) Analysis

In-gel trypsinization of protein spots was performed as previously described [44]. Obtained peptides were loaded on MALDI-TOF plates using ZipTip^®^ Pipette Tips (Merck KGaA, Millipore, Darmstadt, Germany). Tryptic peptides were dissolved in sample solvent containing 0.1% TFA and 50% acetonitrile and mixed with an equal volume of matrix solution containing alpha-cyano-4-hydroxycinnamic acid, 0.1% TFA, and 75% acetonitrile. An amount of 1.5 µL of these mixtures was spotted onto a target plate and analyzed with MALDI-TOF mass spectrometer (Micromass, Waters, Manchester, UK) operated in positive-ion reflectron mode. Obtained spectra were then examined in MASCOT search engine (http://www.matrixscience.com/cgi/search_form.pl?FORMVER=2&SEARCH=PMF). Organisms taxonomically related to bumble bees were selected, as bumble bees are not present in the database. “Other Metazoa” was selected as the taxonomical group, as it is a diverse group including all venomous animals as well as *Drosophila* sp., the taxonomically closest species to bumble bees. This database includes venom toxins from venomous metazoans such as bees (including *B*. *terrestris* and *B*. *impatiens*), spiders, scorpions, and snakes. Both Swissprot and NCBInr databases were used to maximize the number of hits. Hits with MASCOT scores less than 15 were excluded.

## Figures and Tables

**Table 1:** Putative toxins identified in *Thorocabombus* venom. Toxin names and the corresponding 2D gel electrophoresis (2DE) gel spots are indicated. The presence of the identified toxin (and/or its homologs/ortholog) in *B. terrestris* and *A. mellifera* venom is presented.

Putative Toxin Family	Spot	Toxin Name	*B. terrestris*	*A. mellifera*
*Peptidase/protease*	I	Peptidase I *, Aminopeptidase N **	Dipeptidyl peptidase IV, Serine carboxypeptidase, Prolylcarboxypeptidase	Dipeptidyl peptidase IV, Serine carboxypeptidase, Prolylcarboxypeptidase
	IV	Venom protease		
	VII	Serine protease snake	Serine protease 1–6 CUB serine protease	Serine protease snake, CLIP serine protease, CUB serine protease
*Esterase*	II	Venom acid phosphatase Acph1-like	Acid phosphatase	Acid phosphatase 1
	V	Arginine Kinase		
	VI	Phospholipase A_2_	Phospholipase A_2_-1, Phospholipase A_2_-2	Phospholipase A_2_-1, Phospholipase A_2_-2
*Carbohdyrate metabolism*	III	Hyaluronidase 1	Hyaluronidase	Hyaluronidase
*Serine protease inhibitor*	VIII	Kunitz-type serine protease inhibitor	Serpin 1–3	Serpin 1–2

* *B. sylvarum*, ** *B. humilis*.

## Supplementary Materials

The following are available online at www.mdpi.com/2072-6651/9/11/362/s1, Figure S1: 2DE gel images of *Thoracobombus* venom. Representitive images of venom profile of (**A**) *B. humilis*, (**B**) *B. pascuorum*, (**C**) *B. ruderarius*, (**D**) *B. sylvarum*, and (**E**) *B. zonatus*. Gel analyses were conducted in triplicates. Marked spots are spectrometrically identified (See: Tables S3–S7), Table S1: Sample Collection. Locations, number of specimens, and plants species where the bumble bee species are collected from, Table S2: Common and differentially expressed protein spots in *Thoracobombus venom*. Spots intensities are compared in *B. humilis, B. pascuorum*, *B. ruderarius*, *B. sylvarum,* and *B. zonatus* venom, Table S3: List of identified proteins in *B. humilis* venom, Table S4: List of identified proteins in *B. pascuorum* venom, Table S5: List of identified proteins in *B. ruderarius* venom, Table S6: List of identified proteins in *B. sylvarum* venom, Table S7: List of identified proteins in *B. zonatus* venom.
