# Supplementary material for: Proteomic Characterization of the Venom of Five Bombus (Thoracobombus) Species
Source: Toxins (Basel). 2017 Nov 11;9(11):362. doi: 10.3390/toxins9110362 (PMC5705977; doi:10.3390/toxins9110362)
Supplement: Supplementary file 1 [file toxins-09-00362-s001.pdf]

Supplementary Materials: Proteomic Characterization of the Venom of Five *Bombus* (*Thoracobombus*) Species

Nezahat Pınar Barkan, Mustafa Bilal Bayazit and Duygu Ozel Demiralp

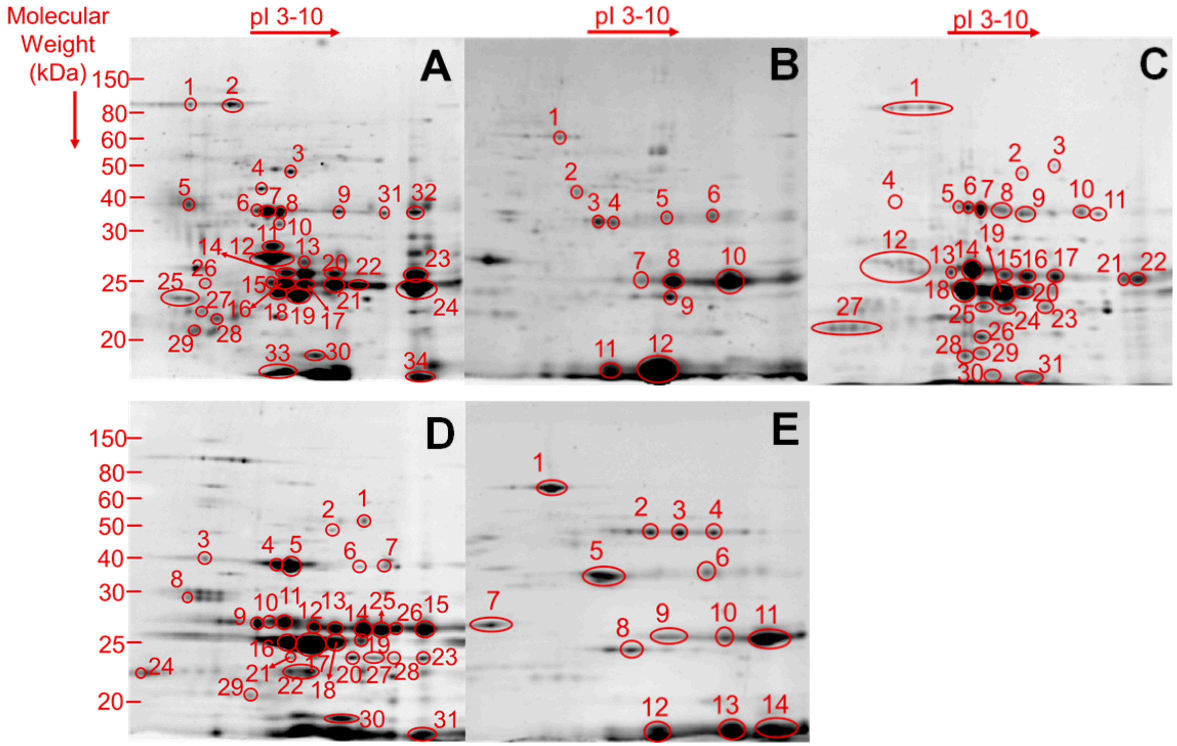

Figure S1. 2DE gel images of *Thoracobombus* venom. Representitive images of venom profile of (**A**) *B. humilis*, (**B**) *B. pascuorum*, (**C**) *B. ruderarius*, (**D**) *B. sylvarum*, and (**E**) *B. zonatus*. Gel analyses were conducted in triplicates. Marked spots are spectrometrically identified (See: Tables S3–S7).

Table S1. Sample Collection. Locations, number of specimens, and plants species where the bumble bee species are collected from.

Species	Location	Date	Altitude (m)	Specimens	Foraged plant species
B. humilis	Ankara-Çerkeş Road, Kızılcahamam-ANKARA 40°33.535′K 32°39.026′D	02/07/2014	1120	16	*Anchusa leptophylla* ROEMER ET SCHULTES subsp. *incana* (LEDEB.) CHAMB., *Stachys byzantina* C. KOCH
	Ankara-Çerkeş Road, Kızılcahamam-ANKARA 40°33.535′K 32°39.026′D	17/07/2014	1120	34	*Anchusa leptophylla* ROEMER ET SCHULTES subsp. incana (LEDEB.) CHAMB., *Melilotus officinalis* (L.) DESR., *Nepeta nuda* L. subsp. *nuda* L., *Stachys byzantina* C. KOCH, *Nepeta nuda* L. subsp. *nuda* L.
	Soğuk Su National Park, Kızılcahamam-ANKARA 40°27.113′K 32°37.355′D	04/07/2014	1216	15	*Trifolium* sp., *Lathyrus incurvus* (ROTH.) WILLD.
B. pascuorum	Abant Lake Natural Park-BOLU 40°36′K 31°16′D	09/08/2013	1350	10	*Carduus nutans*
	Soğuk Su National Park, Kızılcahamam-ANKARA 40°27.113′K 32°37.355′D	04/07/2014	1216	22	*Trifolium* sp., *Lathyrus incurvus* (ROTH.) WILLD.
	Ankara-Çerkeş Road, Kızılcahamam-ANKARA 40°33.535′K 32°39.026′D	02/07/2014	1120	6	*Anchusa leptophylla* ROEMER ET SCHULTES subsp. *incana* (LEDEB.) CHAMB., *Melilotus officinalis* (L.) DESR., *Nepeta nuda* L. subsp. *nuda* L., *Stachys byzantina* C. KOCH, *Nepeta nuda* L. subsp. *nuda* L.
	Ankara-Çerkeş Road, Kızılcahamam-ANKARA 40°33.535′K 32°39.026′D	17/07/2014	1120	3	*Anchusa leptophylla* ROEMER ET SCHULTES subsp. *incana* (LEDEB.) CHAMB. *Melilotus officinalis* (L.) DESR., *Nepeta nuda* L. subsp. *nuda* L., *Stachys byzantina* C. KOCH,
	Ilgaz Mountain National Park-ÇANKIRI 41°4.306′K 33°43.815′D	19/07/2014	1857	19	*Allium* sp., *Trifolium* sp.
	Ilgaz Mountain National Park-ÇANKIRI 41°4.306′K 33°43.815′D	30/08/2014	1857	7	*Epilobium angustifolium* L.
B. ruderarius	Erciyes Mountain-KAYSERİ 38°32.807′K 35°31.427′D	28/07/2014	2177	35	*Carduus* sp.
B. sylvarum	Erciyes Mountain-KAYSERİ 38°32.807′K 35°31.427′D	28/07/2014	2177	43	*Carduus* sp.
B. zonatus	H. U. Beytepe Campus, ANKARA 39°52.321′K 32°44.168′D	24/07/2014	1038	23	*Syringia* sp.
	Ankara-Çerkeş Road, Kızılcahamam-ANKARA 40°20.442′K 32°41.731′D	02/07/2014	991	2	*Salvia candidissima* VAHL subsp. *candidissima* VAHL.
	H. U. Beytepe Campus, ANKARA 39°52.265′K 32°43.844′D	14/07/2014	1017	4	*Consolida regalis* S. F. GRAY subsp. *paniculata* (HOST) SOO var. *paniculata* (HOST) SOO

Table S2. Common and differentially expressed protein spots in *Thoracobombus venom*. Spots intensities are compared in *B. humilis, B. pascuorum, B. ruderarius, B. sylvarum,* and *B. zonatus* venom.

Species	Number of common protein spots	Number of differentially expressed protein spots ($p < 0.05$)
B. humilis/B. pascuorum	56	15
B. humilis/B. sylvarum	67	19
B. humilis/B. ruderarius	66	22
B. humilis/B. zonatus	45	23
B. pascuorum/B. sylvarum	48	14
B. pascuorum/B. ruderarius	46	21
B. pascuorum/B. zonatus	31	16
B. sylvarum/B. ruderarius	61	19
B. sylvarum/B. zonatus	46	18
B. ruderarius/B. zonatus	44	19

Table S3. List of identified proteins in *B. humilis* venom.

Spot #	Access #	Identified protein	Score	Theoretical pI/Mw (kDa)	Observed pI/Mw (kDa)	Sequence coverage (%)	Matched/unmatched peptides
1	XP_013164724	Neurexin-4 isoform X2	55	5.83/144.4	5.63/153.32	8	9/1
2	Q10737	Aminopeptidase N	37	6.47/111.2	6.26/151.29	8	7/2
2	Q01202	Paramyosin	20	5.80/101.9	6.26/151.29	7	5/3
3	XP_003694643	Malate dehydrogenase, cytoplasmic	45	6.92/35.9	7.27/56.08	19	5/5
4	EKC41389	39S ribosomal protein L47, mitochondrial	40	9.55/47.9	6.78/49.48	17	6/3
5	XP_008477265	Mitotic checkpoint serine/threonine-protein kinase BUB1-like	56	8.71/35.3	5.64/45.78	23	7/5
6	XP_003486583	Venom acid phosphatase Acph-1-like isoform X3	96	6.84/46.9	6.86/42.39	18	8/5
7	XP_003486583	Venom acid phosphatase Acph-1-like isoform X3	99	6.84/46.9	6.88/42.39	17	8/4
8	XP_003486583	Venom acid phosphatase Acph-1-like isoform X3	95	6.84/46.9	6.90/42.38	16	8/5
9	P85841	Hyaluronidase 1 (Fragment)	43	8.75/45.2	6.82/42.37	10	3/5
10	XP_011559125	L-lactate dehydrogenase isoform X2	62	6.45/37.6	6.86/42.39	21	7/3
11	XP_011184077	Tropomyosin-1, isoforms 33/34 isoform X26	62	4.74/32.7	6.95/33.79	27	7/1
11	LOC100118569	phytanoyl-CoA dioxygenase, peroxisomal-like	44	5.95/32.6	6.95/33.79	20	4/1
12	LOC102680781	TNF receptor-associated factor 4-like	54	8.33/42.5	6.95/31.21	19	7/4
13	XP_005187259	NADH dehydrogenase [ubiquinone] iron-sulfur protein 3, mitochondrial	43	6.97/30	7.51/30.74	13	4/1
13	XP_005175580	Alpha-tocopherol transfer protein-like	52	5.97/20.5	7.51/30.74	18	4/1
14	P00630	Phospholipase A2	35	7.05/19.6	7.18/28.42	13	5/9
15	P43689	Tropomyosin-2	49	4.58/32.6	7.04/24.78	26	6/5
16	P14208	Arginine kinase	32	6.05/40.3	7.2/26.36	28	8/14
17	Q7M4I3	Venom protease	23	8.85/27.6	7.53/26.58	23	3/7
18	P00630	Phospholipase A2	29	7.05/19.6	7.2/26.36	29	5/17
19	P00630	Phospholipase A2	40	7.05/19.6	7.18/28.42	13	5/9
20	Q9U9J4	Arginine kinase	44	6.39/40.5	7.99/28.25	32	9/11

21	KHJ85713	Triose-phosphate isomerase	53	8.18/27.6	7.99/28.25	31	8/13
22	Q9U9J4	Arginine kinase	44	6.39/40.5	7.99/28.25	13	3/7
23	P00630	Phospholipase A2	22	7.05/19.6	9.40/28.20	15	5/7
23	XP_013096194	Centrosomal protein of 290 kDa-like	56	9.04/27.7	9.40/28.20	34	7/5
23	XP_011493908	Cyclin-dependent kinase inhibitor 1C-like	44	11.15/28.9	9.40/28.20	31	5/7
23	XP_012525274	Phosphoserine phosphatase isoform X1	37	8.26/28	9.87/25.84	31	5/9
24	P00630	Phospholipase A2	42	7.05/19.6	9.87/25.84	14	2/2
25	XP_013309262	Ankyrin repeat protein	66	5.30/28.2	5.44/25.16	32	7/4
25	XP_013147109	Adenylate kinase isoenzyme 1 isoform X2	42	6.64/25.8	5.44/25.16	23	5/6
26	AJO64864	Arginine kinase, partial	40	6.78/22.7	5.88/27.80	26	5/6
27	XP_014236257	Ribose-5-phosphate isomerase	40	5.96/25.9	5.7/22.9	25	5/6
28	AGB85951	Triosephosphate isomerase, partial	66	5.07/15.9	6.08/21.7	36	7/7
29	XP_011191994	Peroxiredoxin-6-like	42	6.30/25.4	5.8/20.03	21	4/2
30	P48816	Lysozyme	34	8.99/16.1	7.65/16.53	29	4/6
30	XP_013144263	probable maleylacetoacetate isomerase 1, partial	52	8.57/25.3	7.65/16.53	25	6/4
31	CEF63756	3-hydroxyacyl-CoA dehydrogenase, C-terminal domain	45	9.05/50.1	8.84/42.25	16	7/4
31	XP_011639815	Bystin	50	8.63/50.4	8.84/42.25	17	8/3
32	O76756	40S ribosomal protein S8	37	10.58/24.1	9.8942.70	31	5/7
32	P00630	Phospholipase A2	35	7.05/19.6	9.89/42.70	11	4/8
33	XP_012798920	25 kDa integral membrane protein	61	8.47/15.5	7.45/15.21	39	5/3
34	Q56FG8	60S ribosomal protein L18	37	11.45/21.8	9.88/15.52	21	4

Table S4. List of identified proteins in *B. pascuorum* venom.

Spot #	Access #	Identified protein	Score	Theoretical pI/Mw (kDa)	Observed pI/Mw (kDa)	Sequence coverage (%)	Matched/ unmatched peptides
1	WP_022965612	nitrogen regulation protein NR(I)	80	5.56/20	6.26/151.29	20	10/3
2	XP_005077830	PREDICTED: kelch-like protein 25	79	6.27/66.8	6.78/49.48	28	13/6
2	P21879	Inosine-5'-monophosphate dehydrogenase	50	6.18/53.1	6.78/49.48	26	10/9
2	Q8Z2E5	L-lactate dehydrogenase	34	6.25/42.9	6.78/49.48	15	4/4
3	XP_003486583	Venom acid phosphatase Acph-1-like isoform X3	88	6.84/46.9	6.86/42.39	18	8/5
4	XP_003486583	Venom acid phosphatase Acph-1-like isoform X3	80	6.84/46.9	6.92/42.38	18	8/5
5	Q8LPJ5	Isocitrate dehydrogenase [NADP], chloroplastic/mitochondrial	50	8.05/54.5	7.09/42.37	15	7/4
5	WP_034695000	transporter	63	6.08/46.8	7.09/42.37	28	9/7
5	WP_023383864	peptide ABC transporter ATP-binding protein	64	9.01/37.4	7.09/42.37	34	9/8
5	P49597	Protein phosphatase 2C 56	38	5.81/47.9	7.09/42.37	13	5/3
5	Q8IY84	Serine/threonine-protein kinase NIM1	59	8.53/49.9	7.09/42.37	20	6/2
5	Q9ZKU5	Acetate kinase	48	6.47/44.9	7.09/42.37	22	7/10
6	P85841	Hyaluronidase 1 (Fragment)	62	8.75/45.2	8.84/42.25	24	6/5
7	Q9U9J4	Arginine kinase	36	6.39/40.5	7.2/26.36	20	6/4

8	Q7M4I3	Venom protease	40	8.85/27.6	7.62/26.58	23	6/4
9	Q6VU70	Apolipophorin-3	23	8.71/20.7	7.88/24.25	24	3/8
9	BAH86593	farnesoic acid O-methyltransferase	60	5.86/31.1	7.88/24.25	23	6/4
10	P00630	Phospholipase A2	20	7.05/19.6	9.88/25.84	17	5/6
11	P55948	Metallothionein	20	8.07/7.2	7.07/15.44	37	2/9
11	P31824	Acyl-CoA-binding protein homolog	19	6.56/9.7	7.07/15.44	34	2/9
12	P55928	Potassium channel toxin alpha-KTx 7.2	21	9.24/4.4	7.45/15.21	77	2/13
12	O96669	Putative potassium channel blocker (Fragment)	20	8.68/5.7	7.07/15.44	31	2/13
12	Q10745	Defensin	21	8.70/4.8	7.07/15.44	58	2/13
12	P41965	4 kDa defensin	25	9.69/4.6	7.07/15.44	42	2/11
12	B8XH50	Amphipathic peptide Tx348	21	9.16/7.7	7.07/15.44	25	2/11
12	Q6JQN2	Peptide BmKn2	21	9.10/7.9	7.07/15.44	24	2/11
12	E4VP07	Venom antimicrobial peptide-6	21	9.10/7.9	7.07/15.44	24	2/11

Table S5. List of identified proteins in *B. ruderarius* venom.

Spot #	Access #	Identified protein	Score	Theoretical pI/Mw (kDa)	Observed pI/Mw (kDa)	Sequence coverage (%)	Matched/ unmatched peptides
1	CEF69244	Globin, structural domain and GPCR, 7TM domain-containing protein	40	9.42/69.3	5.55/136.11	14	8/8
1	XP_001602327	PREDICTED: DNA mismatch repair protein Mlh1	52	6.79/80.8	5.55/136.11	16	9/4
2	KPJ17530	Serine protease snake	60	9.02/38.7	6.99/56.53	20	9/5
2	P29190	Phosphoenolpyruvate carboxykinase [GTP]	31	6.86/70.2	6.99/56.53	11	5/2
3	Q622Z7	G protein-coupled receptor kinase 1	32	8.31/74.2	7.21/56.71	9	5/2
3	H9IWW7	Inactive peptidyl-prolyl cis-trans isomerase shutdown	33	5.84/49.6	7.21/56.71	13	4/3
3	CDS26201	t complex protein 11 protein 1	55	5.30/64.4	7.21/56.71	15	8/3
4	XP_011139073	PREDICTED: glutathione synthetase-like	52	8.03/54.9	5.54/42.25	15	6/1
4	KOC67432	Integrin-linked protein kinase	41	6.86/51	5.54/42.25	14	5/2
5	XP_003486583	Venom acid phosphatase Acph-1-like isoform X3	96	6.84/46.9	6.52/42.15	16	8/3
6	Q5BLY5	Venom acid phosphatase Acph-1	23	5.63/45.5	6.8/41.76	7	3/3
7	XP_013390249	PREDICTED: methylmalonic aciduria type A homolog	75	8.79/48.1	6.7/41.39	25	10/2
7	KHN81636	17-beta-hydroxysteroid dehydrogenase type 6	45	7.82/38.8	6.7/41.39	18	5/3
7	XP_011269785	PREDICT: KH domain-containing...signal trans.-associated pr3-like	42	8.85/48	6.7/41.39	13	5/2
8	P85841	Hyaluronidase 1 (Fragment)	50	8.75/45.2	6.95/41.97	18	6/4
9	KHJ42768	intermediate filament tail domain protein	57	6.37/66.3	7.17/40.91	16	9/1
9	XP_003486187	PREDICT: probable serine/threonine-protein kinase DDB_G0282963	48	9.71/46.1	7.17/40.91	17	7/3
9	KMQ86823	dna primase small subunit	42	7.56/49.3	7.17/40.91	16	7/3
10	XP_013294625	peptidase dimerization domain protein	62	6.04/48.3	8.51/41.61	20	6/0
10	CDJ84573	PAK-box P21-Rho-bind / Ser/thr protein kin.-related dom containing...	42	7.59/65.8	8.51/41.61	13	5/1
10	XP_001120785	PREDICT: zinc finger protein 704-like isoform X1	41	8.45/63.8	8.51/41.61	13	5/1
10	XP_012252555	PREDICT: uncharacterized aarF domain-containing protein kinase 1	61	9.09/60	8.51/41.61	17	7/2

11	XP_013091290	PREDICT: cysteine and histidine-rich protein 1-like	43	6.50/40.5	9.01/40.80	14	5/2
12	P22311	Puff II/9-1 protein	38	5.27/33	5.56/30.42	22	5/5
13	P14208	Arginine kinase	32	6.05/40.3	7.2/26.36	28	8/14
14	P00630	Phospholipase A2	22	7.05/19.6	6.47/28.76	18	3/7
14	P00357	Glyceraldehyde-3-phosphate dehydrogenase	36	6.53/35.9	6.63/28.19	19	5/5
14	P56839	Phosphoenolpyruvate phosphomutase	24	5.51/33	6.63/28.19	15	4/6
15	P00630	Phospholipase A2	19	7.05/19.6	7.23/31.07	10	2/3
15	Q5TXB2	Structure-specific endonuclease subunit SLX1 homolog	24	5.76/32	7.23/31.07	12	3/2
16	P14208	Arginine kinase	37	6.05/40.3	7.78/31.06	20	9/12
16	Q27775	Triosephosphate isomerase	24	6.66/28	7.78/31.06	17	3/5
17	XP_002409028	tyrosine kinase, putative	54	6.05/43.9	8.52/31.06	16	5/0
17	XP_004535104	PREDICTED: dehydrogenase/reductase SDR family member 11	43	8.72/28.1	8.52/31.06	19	4/1
17	CDJ81617	dephospho-CoA kinase	43	8.92/26.7	8.52/31.06	21	4/1
17	P00630	Phospholipase A2	17	7.05/19.6	8.52/31.06	7	2/3
18	XP_013395969	PREDICTED: cytochrome P450 2D14-like	44	6.10/24.4	6.47/26.41	27	5/6
18	XP_013409932	PREDICTED: WD repeat-containing protein 92-like, partial	45	6.64/25.6	6.47/26.41	20	4/3
18	KFM60472	Transposable element Tcb1 transposase, partial	41	7.68/23.6	6.47/26.41	20	5/2
18	P00630	Phospholipase A2	16	7.05/19.6	6.47/26.41	10	2/5
18	A8Y461	Enolase-phosphatase E1	18	5.38/28.2	6.47/26.41	6	2/4
19	Q7M4I3	Venom protease	33	8.85/27.6	7.17/25.96	22	4/3
20	Q61D43	Mediator of RNA polymerase II transcription subunit 18	33	9.15/26.1	8.20/25.97	23	7/14
20	XP_003386765	PREDICTED: glutathione S-transferase-like	43	6.23/23.2	8.20/25.97	36	7/9
20	XP_005190650	PREDICTED: glutaredoxin 3	74	4.83/23.5	8.20/25.97	38	6/2
21	P00630	Phospholipase A2	21	7.05/19.6	9.20/25.97	13	3/7
21	P00630	Phospholipase A2	24	7.05/19.6	9.20/25.97	12	3/2
22	AKS40350	glutathione S-transferase theta 1 [Chilo suppressalis]	47	8.85/26.6	9.39/29.43	26	6/4
23	P00630	Phospholipase A2	37	7.05/19.6	8.41/25.96	20	4/3
23	P10737	Venom allergen 5.02 (Fragment)	17	9.13/24.7	8.41/25.96	13	2/4
24	EFN83275	Inositol monophosphatase	63	5.42/28.8	7.18/25.90	21	6/2
25	XP_011298725	PREDICTED: FK506-binding protein 2 isoform X1	54	4.67/24.6	6.90/25.92	26	6/5
26	A8XHX1	Tetratricopeptide repeat protein 36 homolog	20	5.26/19.8	6.88/24.85	22	3/11
26	Q16981	Neuronal calcium sensor 1	20	4.78/22.2	6.88/24.85	21	3/8
27	AGB86884	proteasome subunit, partial	42	4.42/19.6	4.22/24.90	24	4/4
28	E7CLP0	Putative alpha-neurotoxin RjAa9 (Fragment)	26	8.15/7.6	6.48/21.42	19	2/3
29	P86403	Neurotoxin MeuNaTx-2	23	8.19/7.6	6.85/21.45	21	2/6
30	KMQ93222	protein phosphatase 1l-like protein, partial	55	5.63/12	6.87/18.72	55	5/6
31	KMQ93222	protein phosphatase 1l-like protein, partial	74	5.63/12	7.78/18.70	55	5/1

Table S6. List of identified proteins in *B. sylvarum* venom.

Spot #	Access #	Identified protein	Score	Theoretical pI/Mw (kDa)	Observed pI/Mw (kDa)	Sequence coverage (%)	Matched/unmatched peptides
1	CDJ88047	Dehydrogenase domain containing protein	42	6.08/48.3	8.1/63.38	10	4/7
1	XP_013391792	PREDICTED: uncharacterized protein LOC106159893	33	8.70/64	8.1/63.38	9	4/7
2	KPJ17530	Serine protease snake	30	9.02/38.7	7.27/56.08	9	3/7
2	XP_013081819	PREDICT: MAP kinase kinase kinase 1-like isoform X1	30	5.48/42.1	7.27/56.08	9	3/7
3	CAX82492	Malate dehydrogenase	31	7.59/42	5.64/45.78	12	3/8
4	XP_003486583	Venom acid phosphatase Acph-1-like isoform X3	96	5.33/46.2	6.86/42.39	10	9/4
5	XP_003486583	PREDICTED: venom acid phosphatase Acph-1-like isoform X3	86	6.84/46.9	7.52/42.64	18	9/15
6	P85841	Hyaluronidase 1 (Fragment)	55	8.75/45.2	8.06/42.37	10	3/5
7	EJW83419	hypothetical protein WUBG_05671	67	5.41/42.3	8.84/42.25	20	6/4
8	EGT37222	hypothetical protein CAEBREN_12699	50	6.65/40.2	5.33/30.08	20	6/3
9	P86699	Arginine kinase (Fragments)	19	7.74/25.4	6.66/27.01	6	2/12
10	XP_011873210	PREDICT: isochorismatase domain-containing pr. 2, mitochondrial-like	47	6.30/21.7	6.66/27.01	29	4/12
10	P86699	Arginine kinase (Fragments)	19	7.74/25.4	6.66/27.01	6	2/12
11	EFN72991	FAD synthetase	32	8.67/34.2	6.97/26.55	15	3/10
11	XP_011563998	PREDICTED: peroxisome biogenesis factor 10	33	8.23/30.8	6.97/26.55	17	3/10
12	Q7M4I3	Venom protease	33	8.85/27.6	7.45/26.02	25	3/6
13	KHN71023	Aspartyl protease inhibitor, partial	61	8.62/20.9	7.97/26.07	30	5/12
13	P30114	Glutathione S-transferase class-mu 28 kDa isozyme	17	6.56/24	7.97/26.07	13	2/18
13	P39673	Allergen Mag (Fragment)	23	6.92/39.6	7.97/26.07	11	3/12
14	Q1EIQ3	Peptidase 1	33	6.95/36.6	8.36/26.1	26	6/14
15	XP_011142752	PREDICTED: serine/threonine-protein kinase ICK isoform X2	65	9.34/66.2	9.89/25.84	20	7/11
15	P00630	Phospholipase A2	28	7.05/19.6	9.89/25.84	16	3/13
16	P00630	Phospholipase A2	31	7.05/19.6	7.04/24.78	25	5/10
17	P00630	Phospholipase A2	35	7.05/19.6	7.39/24.65	27	4/8
17	ACD01564	Dopa decarboxylase, partial	63	9.05/21.7	7.39/24.65	32	6/4
17	P00630	Phospholipase A2	35	7.05/19.6	7.39/24.65	31	5/10
18	P46419	Glutathione S-transferase	20	6.32/25.8	7.88/24.25	21	3/7
19	P00630	Phospholipase A2	21	7.05/19.6	8.34/24.77	15	3/6
19	XP_011564135	PREDICTED: transcription factor Sox-14	52	9.94/25.5	8.34/24.77	35	6/2
20	D2Y2Q2	Kunitz-type serine protease inhibitor hainantoxin-XI-13	27	9.36/10.1	8.21/22.04	36	3/8
20	P00630	Phospholipase A2	20	7.05/19.6	8.21/22.04	9	3/8
21	Q5MGE6	Defense protein 3	22	5.75/25	6,8/22,67	22	3/8
22	XP_011666262	PREDICTED: ankyrin-1-like	50	5.94/33	7.02/21.36	34	7/6
23	Q3YAU5	Phospholipase A2 large subunit (Fragment)	18	7.46/11.9	9.89/22.02	29	2/8
24	Q8T6C4	Thioredoxin peroxidase	44	5.78/21.7	4.94/20.63	41	5/8
25	Q1EIQ3	Peptidase 1	34	6.95/36.6	8.74/26.21	26	6/12
26	Q9NH49	Arginine kinase	35	6.19/40.6	9.03/26.14	23	6/7
27	KII66870	Serine/threonine-protein phosphatase 2B catalytic subunit γ isoform	42	9.67/16.4	8.38/23.15	42	6/11

28	P51898	NADH-ubiquinone oxidoreductase chain 4 (Fragment)	31	8.92/25.1	8.51/23.95	24	4/7
29	KFM62790	Endoribonuclease Dicer, partial	40	6.32/20.8	5.94/18.19	29	5/5
30	P00630	Phospholipase A2	42	7.05/19.6	7.98/.34	40	5/13
31	D2Y2Q2	Kunitz-type serine protease inhibitor hainantoxin-XI-13	67	9.36/10.1	9.89/15.37	27	3/4

Table S7. List of identified proteins in *B. zonatus* venom.

Spot #	Access #	Identified protein	Score	Theoretical pI/Mw (kDa)	Observed pI/Mw (kDa)	Sequence coverage (%)	Matched/unmatched peptides
1	A8XFM8	Lon protease homolog, mitochondrial	26	6.49/107.1	6.26/151.29	11	8/6
2	EFN76202	Arginyl-tRNA synthetase, cytoplasmic	44	6.85/78.8	7.26/49.41	9	6/0
2	XP_011050254	PREDICTED: putative glycerol kinase 5 isoform X3	55	8.71/52.6	7.26/49.41	14	6/0
2	XP_012150740	PREDICTED: zinc finger protein 37 homolog isoform X14	42	6.15/55	7.26/49.41	11	5/1
2	CDW54939	acyl coenzyme A dehydrogenase, C 4 to	42	8.38/46.8	7.26/49.41	13	5/1
2	XP_011699966	PREDICTED: catalase isoform X2	47	8.11/55.7	7.26/49.41	8	5/1
3	Q17GM7	Probable citrate synthase 1, mitochondrial	30	8.91//51.8	7.47/49.21	14	5/4
3	XP_012216101	PREDICTED: ribosomal RNA-processing protein 8	60	10.03/55.9	7.47/49.21	17	8/3
4	P85841	Hyaluronidase 1 (Fragment)	55	8.75/45.2	7.72/49.01	19	6/4
5	XP_003486583	Venom acid phosphatase Acph-1-like isoform X3	78	6.84/46.9	6.95/33.79	15	3/5
6	Q6R7M4	Methyl farnesoate epoxidase	31	8.78/56.9	7.92/36.36	15	8/6
7	XP_011647527	PREDICTED: caspase-1-like	40	5.48/30.6	5.52/29.58	26	5/6
7	Q61UX1	Nascent polypeptide-associated complex subunit alpha	33	5.01/22	5.52/29.58	34	4/7
7	XP_002433504	protease m1 zinc metalloprotease, putative	41	5.50/26.3	5.52/29.58	24	4/4
7	O16127	Tropomyosin A (Fragment)	20	4.60/32.2	5.52/29.58	10	3/5
8	XP_008192565	PREDICTED: 39S ribosomal protein L28, mitochondrial	45	9.19/31.9	7.97/26.07	30	6/6
8	KKA73268	tRNA synthetase, partial	53	10.45/27.4	7.97/26.07	31	6/4
9	Q7M4I3	Venom protease	28	8.85/27.6	9.89/25.84	17	3/4
10	P86699	Arginine kinase (Fragments)	38	7.74/25.4	8.36/26.1	33	8/13
11	P00630	Phospholipase A2	20	7.05/19.6	9.89/25.84	13	3/8
12	M1J7U4	Putative sodium channel alpha-toxin Acra5	28	7.64/8.1	7.07/15.44	46	3/10
12	KFB53291	Methionine--tRNA ligase	59	9.34/9.7	7.07/15.44	53	5/8
12	Q1HPL8	NADH dehydrogenase [ubiquinone] 1 beta subcomplex subunit 10	31	5.93/19.2	7.07/15.44	27	4/9
12	P56219	Potassium channel toxin alpha-KTx 4.2	19	8.94/6.7	7.07/15.44	31	2/11
13	CDQ08235	Protein Bm12574, partial	72	4.78/6.9	7.45/15.21	90	6/6
13	P00038	Cytochrome c	23	9.50/12	7.45/15.21	33	3/9
13	Q8T3S7	Kunitz-type serine protease inhibitor U1-aranetoxin-Av1a	22	9.11/9.6	7.45/15.21	30	2/4
14	KFM80435	Coiled-coil-helix-coiled-coil-helix domain-containing protein 7, partial	50	8.84/12.5	9.89/15.37	44	4/2
14	XP_014253312	PREDICTED: leucine-rich repeat-containing protein 28 isoform X3	79	9.11/31.1	9.89/15.37	29	7/2
